# (Metallo)porphyrins for potential materials science applications

**DOI:** 10.3762/bjnano.8.180

**Published:** 2017-08-29

**Authors:** Lars Smykalla, Carola Mende, Michael Fronk, Pablo F Siles, Michael Hietschold, Georgeta Salvan, Dietrich R T Zahn, Oliver G Schmidt, Tobias Rüffer, Heinrich Lang

**Affiliations:** 1 Solid Surfaces Analysis Group, Institute of Physics, Faculty of Natural Sciences, TU Chemnitz, D-09107 Chemnitz, Germany; 2 Inorganic Chemistry, Institute of Chemistry, Faculty of Natural Sciences, TU Chemnitz, D-09107 Chemnitz, Germany; 3 Semiconductor Physics, Institute of Physics, Faculty of Natural Sciences, TU Chemnitz, D-09107 Chemnitz, Germany; 4 Material Systems for Nanoelectronics, TU Chemnitz, D-09107 Chemnitz, Germany; 5 Institute for Integrative Nanosciences, IFW Dresden, Helmholtzstrasse 20, 01069 Dresden, Germany

**Keywords:** atomic force microscopy, magneto-optical Kerr effect spectroscopy, scanning tunnelling microscopy and spectroscopy, self-assembly, surface-confined 2D polymerization, transport properties

## Abstract

The bottom-up approach to replace existing devices by molecular-based systems is a subject that attracts permanently increasing interest. Molecular-based devices offer not only to miniaturize the device further, but also to benefit from advanced functionalities of deposited molecules. Furthermore, the molecules itself can be tailored to allow via their self-assembly the potential fabrication of devices with an application potential, which is still unforeseeable at this time. Herein, we review efforts to use discrete (metallo)porphyrins for the formation of (sub)monolayers by surface-confined polymerization, of monolayers formed by supramolecular recognition and of thin films formed by sublimation techniques. Selected physical properties of these systems are reported as well. The application potential of those ensembles of (metallo)porphyrins in materials science is discussed.

## Review

### Introduction

Macrocyclic compounds occurring in nature play an essential role in biological and chemical processes, whereby porphyrins and their corresponding metal species rank among the most frequently occurring and important representatives [1–3]. In addition, there is a plethora of synthetic possibilities to functionalize (metallo)porphyrins in order to better match their physical properties to requirements of the envisaged application [1–3]. Because of the general high chemical and thermal stability, thin films of (metallo)porphyrins can be obtained by using organic molecular beam deposition (OMBD) techniques, which enable a better potential to build molecular spintronic devices [4–9].

A crucial step and thus, a prerequisite for the reliable implementation of (metallo)porphyrin-based thin films in a device, is the understanding of the electrical response and local transport properties [10]. However, thorough investigations of the latter properties, also in dependence of the thin film morphology, are still missing. Since magneto-optical effects are used in various optoelectronic devices [11], the optical characterization of thin films of (metallo)porphyrins by means of spectroscopic magneto-optical Kerr effect (MOKE) measurements would be very interesting.

Moreover, as MOKE measurements allow one to address magnetic properties with both high sensitivity and high spatial resolution, magneto-optical techniques in the visible or X-ray photon energy range have been supposed to be important for the integration of single-molecule magnets into spintronic or quantum computing devices [12]. For the design of such devices the knowledge of the photon energy at which the MOKE is largest in magnitude is of crucial importance.

The number of reports on spectroscopic MOKE investigations are very limited, while magneto-optical studies of porphyrinoids [13] conducted by magnetic circular dichroism (MCD) are numerous and have been already comprehensively reviewed [14]. As the commonly applied MCD spectroscopy requires the compounds to be solubilized or deposited on transparent substrates, spectroscopic MOKE measurements are not limited by such prerequisites. To the best of our knowledge there are, besides our own contributions, no reports available on spectroscopic MOKE measurements of either multimetallic complexes [15] or porphyrinoids [16–19], although MOKE magnetometric investigations have been already reported for multimetallic complexes [20–21].

For potential applications it is necessary that thin films and/or (sub)monolayers of (metallo)porphyrins can be fabricated with a high degree of reproducibility. One possibility to achieve controlled ordering on surfaces (beyond lithography [22–23]) is to functionalize the (metallo)porphyrins with terminal groups that allow their self-assembly on surfaces. Self-assemblies, giving rise to well-defined long-range ordered lateral structures, are frequently reported [24–28]. For example, in case that (metallo)porphyrins were functionalized with terminal hydroxyl groups they self-assemble on surfaces through the formation of hydrogen bonds [29–33]. This promising approach has, however, the disadvantage that the 2D networks lack stability as their mutual interactions are noncovalent and thus weak in nature. It would be thus fascinating to create covalently bonded ensembles on surfaces, or, even more challenging, to induce a 2D surface polymerization. Despite the difficulties to control covalent bond formation on surface, a small number of such studies already exist [34–39], including the first example of a 2D surface polymerization of porphyrins [40–42]. In the latter case, the porphyrins were functionalized with halides, preferably –Br or –I, to enable surface-confined C,C cross-coupling reactions on metallic substrates based on, for example, the Ullman coupling reaction [40–42].

**Scheme 1:**
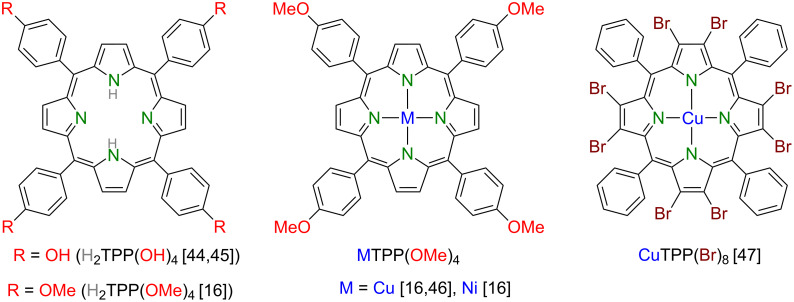
Chemical structures of (metallo)porphyrins under review here.

Here, we report on the multifaceted use of appropriate (metallo)porphyrins (Scheme 1) to form (dendritic) thin films, (sub)monolayers and nano-ribbons and describe subsequently performed physical studies of those ensembles. We like to emphasize that the molecular interface formed of mostly (metallo)phthalocyanines as the organic part and different inorganic materials has been the explored with respect to its application potential in a comprehensive recent review [43].

### Results

**Local electrical transport characteristics and morphology characteristics of nanostructured CuTPP(OMe)_4_ on Ni substrates** [46]**:** Tailored (metallo)porphyrins present a rich potential for device integration. From the point of view of synthesis, well-developed methodologies allow for the preparation of a variety of functionalized porphyrins. This is essential in order to produce, for example, macromolecules for particular applications by precisely tailoring adequate substituents [48–49]. Besides the high potential as building blocks for molecular spintronic devices [4,6,8,50], (metallo)porphyrins open new venues for the preparation of functional nano-architectures [51–56] that offer novel alternatives for device approaches such as sensors or organic field-effect transistors (OFETs) [57]. The compatibility of (metallo)porphyrin compounds with deposition techniques of organic molecules presents a great potential for the implementation and scaling down of porphyrins into current device fabrication processes, where thin films are required. Therefore, the understanding of the electrical transport properties of (metallo)porphyrin compounds (down to the nanoscale) is a crucial step for a reliable implementation in devices [10]. When performed at the nanoscale level, for example via spectroscopic techniques such as conductive atomic force microscopy, the easy correlation between morphology and electrical properties delivers valuable insights that contribute to elucidate possible microscopic mechanisms that determine the electrical performance of organic devices. Here, dominant transport mechanisms or local defect-driven conductive domains can be revealed.

**Figure 1:**
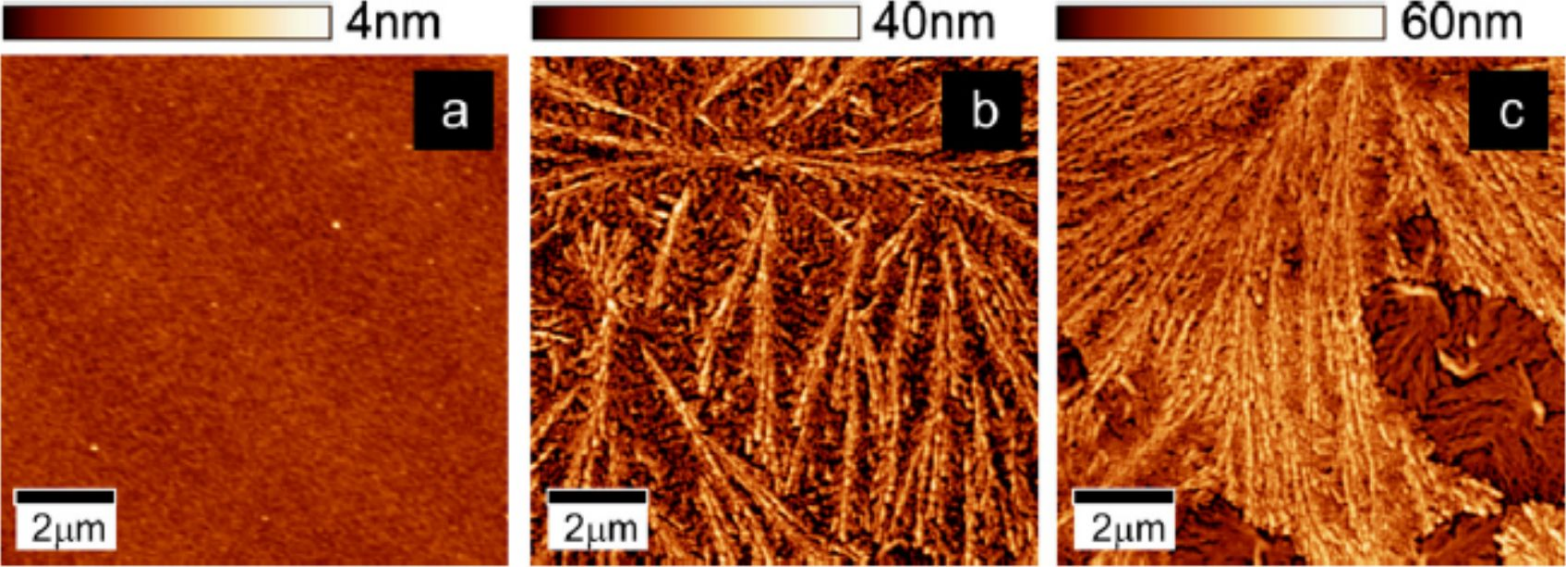
Surface topography determined by AFM as a function of thickness. Cu-TMPP (= CuTPP(OMe)_4_) (a) 35 nm, (b) 82 nm, and (c) 117 nm thick. Organic films are deposited on a 30 nm thick Ni substrate. Once the organic aggregation is initialized, the sample surface of Cu-TMPP films evolves from an aleatory filamentary distribution (b) to a layered filamentary organization (c). Reproduced with permission from [46], copyright 2014 Elsevier.

**Figure 2:**
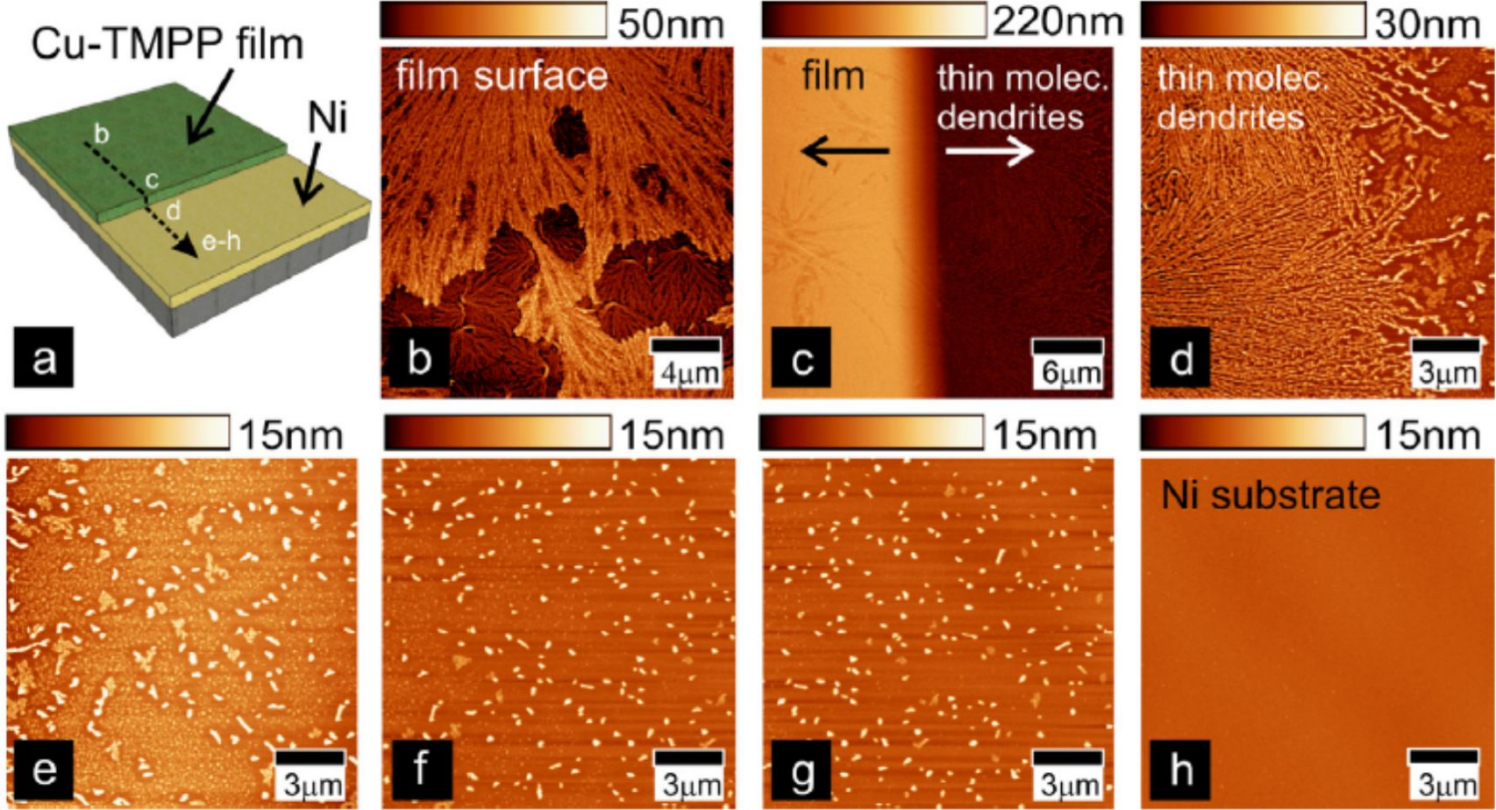
Formation of molecular dendrites in a 117 nm thick Cu-TMPP (= CuTPP(OMe)_4_) sample at different regions along the surface. (a) Diagram of the sample structure. AFM topography characteristics: (b) on top of the organic film, (c) at the edge, and (d–h) at different areas moving away from the edge. As the molecular dendrites disappear, the bottom Ni substrate (0.4 nm rms) becomes visible (h). Reproduced with permission from [46], copyright 2014 Elsevier.

For the latter reason we aimed to investigate the local transport characteristics of films of CuTPP(OMe)_4_. Three thin films of CuTPP(OMe)_4_ with thicknesses of 35, 82, and 117 nm (Figure 1) were deposited by OMBD (pressure 2 × 10^−7^ mbar, deposition rate 5 Å/min, temperature 325 °C) on a 30 nm thick nickel bottom electrode on top of a Si(100)/SiO_2_ wafer (Figure 2). Here, Ni substrates were selected for the growth of CuTPP(OMe)_4_ in order to investigate a system that may possess valuable possibilities for future device applications, in which the implementation of ferromagnetic substrates would enable interesting physical phenomena such as spintronic capabilities. On the other hand, the possibility of choosing different thicknesses of CuTPP(OMe)_4_ allows for a quantitative investigation of the transport properties in order to identify dominant transport mechanism of the organic material. A shadow mask during deposition was employed to avoid additional photolithography processing. This shadow mask also allows for the formation of thin molecular dendrites and even single dendrites on the Ni surface (Figure 2). The growth conditions of the dendrites were investigated in detail, as indicated in Figure 1 and Figure 2. For example, height and length of the dendrites increase in dependence of the film thicknesses (Figure 1). We could show that by adequate optimization of parameters of the thin film formation the fabrication of filamentary nanostructures with predefined dimensions seems possible. Related structural aggregates were hitherto obtained through solution processing only [58]. The disadvantages of this wet-chemistry deposition technique are the possible contamination and/or substrate oxidation [11,15,59]. The dendrites could be particularly suitable to match certain device requirements. For example, after proper manipulation and lithography procedures single molecular nanowires or dendrites might be realized. This certainly opens possibilities for application in materials science, for example, the integration into molecular-based devices. In order to investigate this in more detail, we performed current-sensing atomic force microcopy (cs-AFM) studies of thin films of CuTPP(OMe)_4_ deposited an Ni substrates.

**Figure 3:**
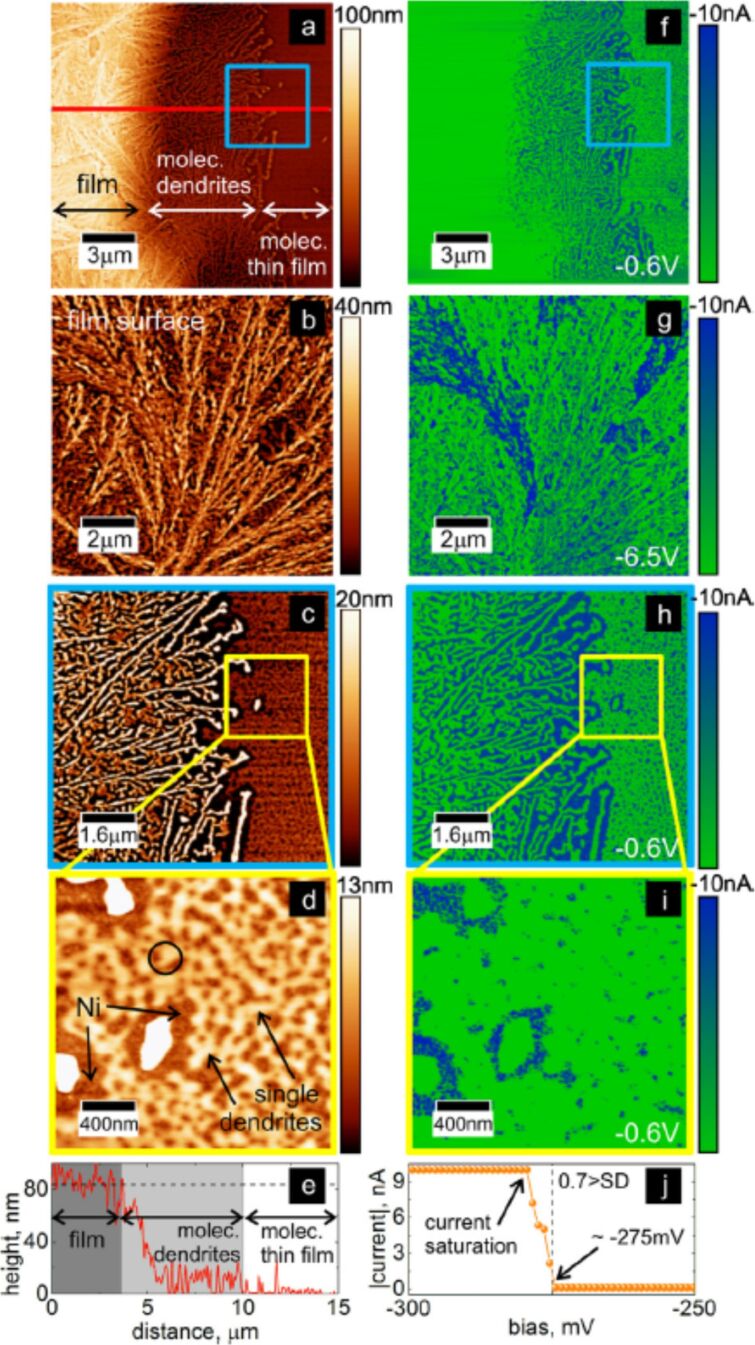
Transport in Cu-TMPP (= CuTPP(OMe)_4_) films and dendrites, (a–d) AFM topography characteristics. The darker areas correspond to the Ni substrate below the molecular dendrite structures (brighter features), (f–i) cs-AFM current maps for the same areas indicated in a–d, (e) height–distance profile as indicated by the solid red line in (a), showing the organic film, thin molecular dendrites film, and single dendrites regions. The dotted line indicates the average height of the organic film, (j) local *I*–*V* characteristics for a single dendrite (3–4 nm height) as indicated with a black circle in (d), the *I*–*V* data represent an average of five consecutive cycles. The highest standard deviation (SD) for data points corresponds to 0.7. (For interpretation of the references to color in this figure caption, the reader is referred to the web version of this article.) Reproduced with permission from [46], copyright 2014 Elsevier.

The electrical characteristics of the CuTPP(OMe)_4_ thin film with 82 nm thickness determined via a cs-AFM study is shown in Figure 3. As the thin film topography and conductivity were acquired simultaneously the correlation of both features is straightforward. The electrical response of the different organic films appears to be reversible, which suggests that the structural integrity of the molecules is preserved after the electrical measurements [46]. Despite of this, due to the molecular aggregation and dendrite-layered organization of the films, which may induce interface defects, a local inhomogeneity of the electrical transport was detected. The distribution of conducting sites on the organic surface and a transport regime were identified. The transport exhibited a transition from an ohmic (linear) to an exponential conductive regime. The utilization of different thicknesses of CuTPP(OMe)_4_ provided a proper calibration between the amount of material that is evaporated and the actual dimensions of the organic filaments. This offers valuable information in order to obtain single organic filaments that could eventually play a role as organic nanowires (Figure 2d). cs-AFM studies showed to be suitable to obtain quantitative measures of local transport properties in relation to topographical features and this approach could be applied to other kind of molecular systems, e.g., (metallo)porphyrins with different metal centers. This leads to the control and tuning of the electrical properties, where the interaction and coupling of the metal centers, the molecule and the substrate would play an important role on the transport properties of (metallo)porphyrin-based devices. We concluded that such studies would allow one to find suitable (metallo)porphyrins to explore interesting fields such as spintronic applications by coupling for example ferromagnetic substrates with suitable porphyrin structures. Consequently, we started to synthesize two new series of (metallo)porphyrins for related studies in which the metal centers and the terminal groups were varied [60]. Unfortunately, all of these (metallo)porphyrins were not suited for OMDB as they all thermally decomposed before sublimation (see below).

**Optical and magneto-optical characterization of thin films of H_2_TPP(OMe)_4_, NiTPP(OMe)_4_ and CuTPP(OMe)_4_** [16]**:** Thin films of the porphyrins under review were deposited by OMBD (pressure ca. 1 × 10^−4^ Pa, deposition rates of 5–10 Å/min). For the deposition of NiTPP(OMe)_4_ and CuTPP(OMe)_4_ sublimation temperatures of 300–310 °C were required, whereas H_2_TPP(OMe)_4_ already sublimated with a stable deposition rate at 295 °C [16]. This might be attributable to the lower molecular weight of H_2_TPP(OMe)_4_ or, more likely, to a different kind of intermolecular interactions. It is this specific finding which prompted us to replace the terminal -N(iPr)_2_ by –NMe_2_ groups as described in [60]. However, the lower molecular weight and the potentially different intermolecular interactions did not enable the deposition of the –NMe_2_-terminated (metallo)porphyrins. As substrates Si(111) wafers covered with a native oxide layer (2 nm) and silicon pieces covered with 100 nm thick Au layers were applied.

**Figure 4:**
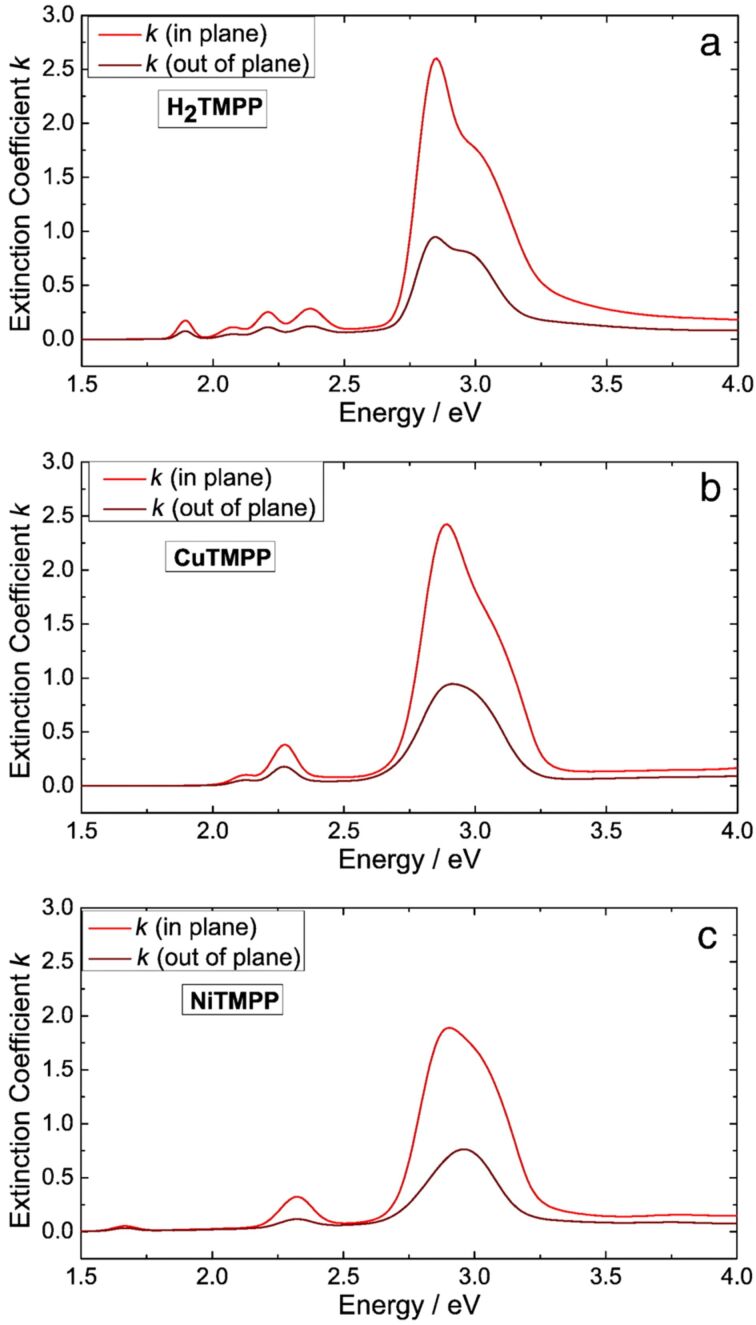
Extinction coefficient *k* for a) H_2_TMPP, b) CuTMPP and c) NiTMPP. The uniaxial anisotropic model results in different intensities in- and out-of-plane. Reproduced with permission from [16], copyright 2014 Elsevier. Notice: H_2_TMPP, CuTMPP and NiTMPP refer to H_2_TPP(OMe)_4_, NiTPP(OMe)_4_ and CuTPP(OMe)_4_, respectively, as displayed in Scheme 1.

Variable angle spectroscopic ellipsometry (VASE) was used to determine the absorption spectra of the as-obtained thin films. The extinction coefficients of the thin films display the typical features expected for (metallo)porphyrins, including expected differences in the number of absorption bands due to symmetry considerations regarding free-base H_2_TPP(OMe)_4_ compared to metalated NiTPP(OMe)_4_ and CuTPP(OMe)_4_ [16–17]. Additionally, the optical anisotropy of the extinction coefficient determined from VASE in the spectral range of the first absorption band (see Figure 4 for the in plane-and out-of-plane extinction coefficient) allowed for the determination if the molecular tilt angle α, that is, the angle of the deposition molecules with respect to the substrate.

As the interaction of the (metallo)porphyrins with metallic surfaces is stronger than that with semiconductor surfaces, the angle α of all three different types of compounds was expected to be smaller on Au compared to Si substrates. However, the opposite observation was made. For each investigated compound the angle α on Si is ca. 3° smaller than that on Au substrates. This is attributed to roughness effects. VASE measurements are thus excellently suited to determine the orientation of (metallo)porphyrins in thin films with respect to the substrate surface. Furthermore, the substrate smoothness was shown to have an impact on the orientation of the (metallo)porphyrins with respect to the substrate surface. Different values of α might be one influencing factor determining the performances of porphyrin-based devices. The molecule–substrate interaction dictates the orientation of the first molecular layer. The orientation of this first layer strongly influences the orientation of the next layers. The templating effect of the first layer can extend up to tens of nanometers in phthalocyanine layers [61].

In subsequent aging studies and in combination with near edge X-ray absorption fine structure (NEXAFS) spectroscopy we could show that the values of α apparently increase by 4–7° on both substrates over a period of nine months. A long-term study showed that also the optical spectra of H_2_TPP(OMe)_4_ change after ageing in air. Not only the ratio of in-plane to out-of-plane extinction coefficient changes with time, but also the roughness of the samples stored in air increases more rapidly leading to an increase of the nominal film thickness (both determined from the ellipsometry data). This could be explained considering that oxygen or moisture from the atmosphere diffuses into the H_2_TPP(OMe)_4_ film progressively, with a slight saturation tendency after 20 days. Increasing roughness might also be a sign of crystallization induced by oxygen or moisture [62]. The origin of these changes might be attributed to (re)crystallization processes. Nevertheless, this study confirmed that long-stime stability studies of any devices based on (metallo)porphyrins are required.

**Figure 5:**
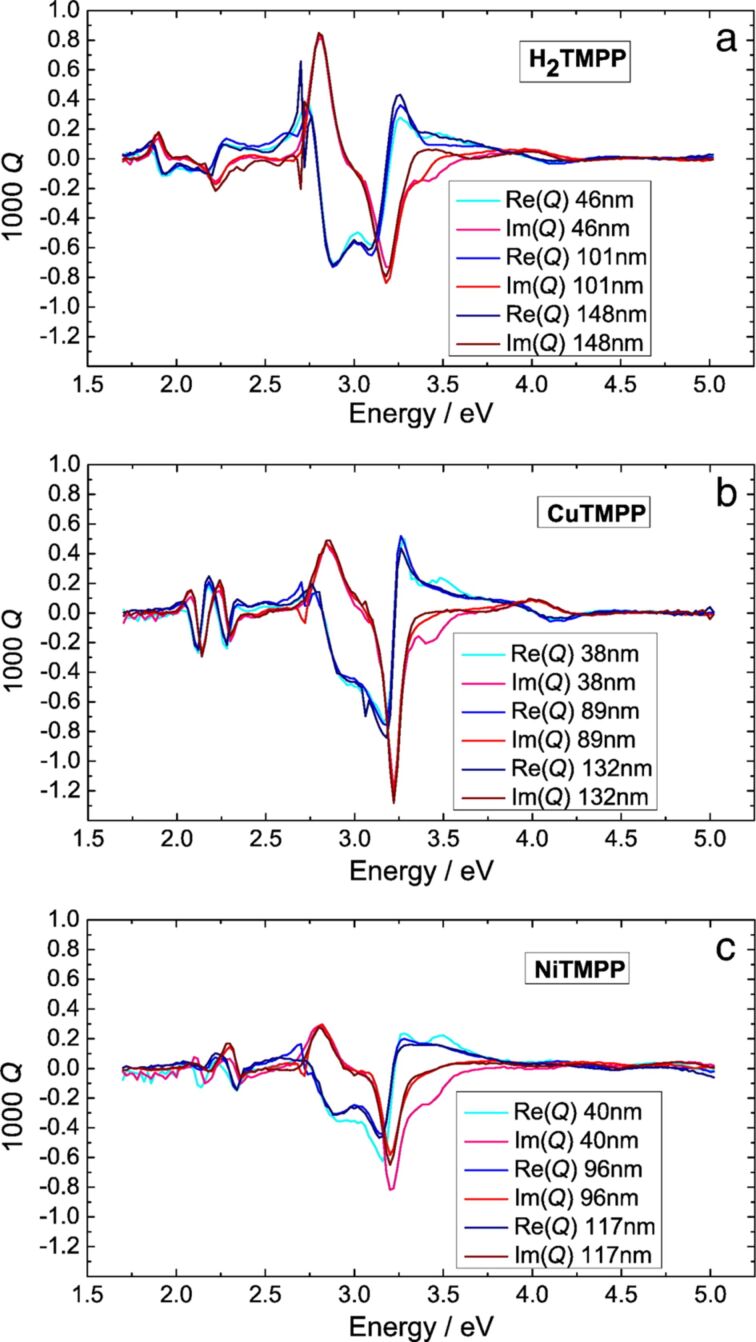
Energy dispersion of the magneto-optical Voigt constant *Q* for a) H_2_TMPP, b) CuTMPP and c) NiTMPP. For reasons of clarity, *Q* was multiplied by 10^3^ and normalized to a magnetic field of 1 T. Reproduced with permission from [16], copyright 2014 Elsevier. Notice: H_2_TMPP, CuTMPP and NiTMPP refer to H_2_TPP(OMe)_4_, NiTPP(OMe)_4_ and CuTPP(OMe)_4_, respectively, as displayed in Scheme 1.

MOKE spectra were successfully acquired both on the diamagnetic (H_2_TPP(OMe)_4_) and paramagnetic (NiTPP(OMe)_4_ and CuTPP(OMe)_4_) molecular films on silicon substrates. Based on numerical calculations using an optical model described in [18], which was developed by us and applied successfully for porphyrinoid-based thin films [17–18], it was possible to determine the energetic dispersion of the magneto-optical Voigt constant, which is independent of the film thickness (Figure 5).

**Figure 6:**
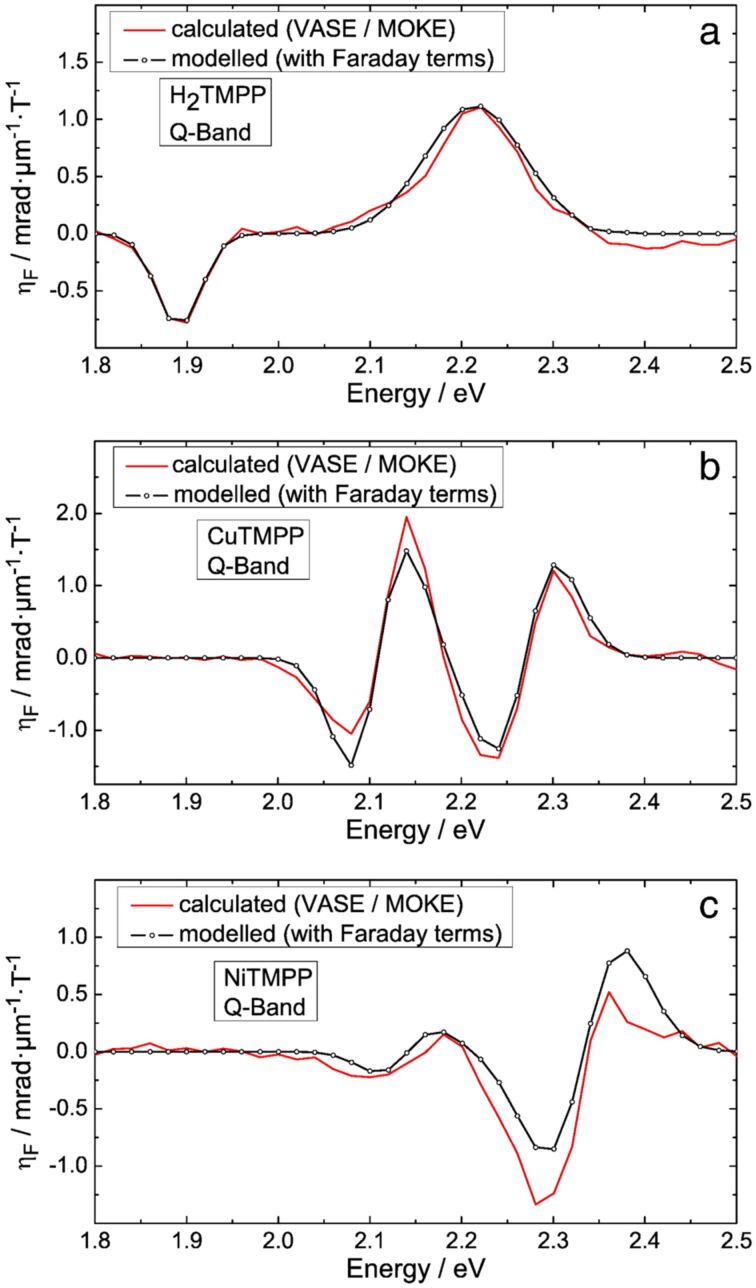
MCD (η_F_) spectra in the Q-band region for a) H_2_TMPP, b) CuTMPP and c) NiTMPP and the modelling with Faraday terms. Reproduced with permission from [16], copyright 2014 Elsevier. Notice: Here mentioned H_2_TMPP, CuTMPP and NiTMPP refer to H_2_TPP(OMe)_4_, NiTPP(OMe)_4_ and CuTPP(OMe)_4_, repectively, as displayed in Scheme 1.

Despite its energy dispersion, the complex magneto-optical constant *Q* is commonly described in literature as the Voigt constant because its material specificity. If known, the Voigt constant can be used to predict the magneto-optical response of a sample both in reflection (MOKE geometry) and transmission (Faraday geometry) measurements. The ellipticity of the transmitted light is labelled as magnetic circular dichroism (MCD). For solutions or transparent samples MCD spectroscopy is often carried out in combination with UV–vis spectroscopy to characterize the electronic properties of molecules, including porphyrinoids. For a better comparison with the existing literature about the magneto-optical response of porphyrinoids we calculated from the Voigt constant the MCD spectra of the investigated molecules. The predicted spectra are shown in Figure 6 (continuous lines) along with the corresponding fitted curves (line plus symbol).

The fine structure of the MCD spectra can be used to extract information about the degeneracy of electronic states, in addition to what is easily readable in the UV–vis spectra [14]. The reason for the fine structure of the MCD spectra and hence for the higher sensitivity to the electronic properties lies in the Zeeman splitting of degenerate electronic levels induced by an external magnetic field. In addition to the energetic position of the spectral features, their line shape observed in the MCD spectra is important for the interpretation of the spectra. The typical line shapes observed in various MCD spectra can be classified as the Faraday A-, B- and C-terms [14].

In the following, we will focus our discussion on the spectral range of the Q band. In the case of H_2_TPP(OMe)_4_ the MCD spectrum in Figure 6a shows only one B-term pair. The energies of the two features of opposite sign correspond to the first and third absorption peak of the UV–vis absorption spectrum. According to [14] the observation of a B-term pair is an indication that no degenerate energy states are involved in optical transitions probed. In this case, the fine structure of the band can rather be explained as a consequence of a coupling between vibrational and electronic states, i.e., vibronic coupling [63]. Compared to the MCD spectra of H_2_PP (without methoxy groups), the line shape is similar but the amplitude of the bands is reduced, indicating that the methoxy groups thus do not suppress the vibronic coupling. For modelling the Q band of NiTPP(OMe)_4_ and CuTPP(OMe)_4_ in the MCD spectra, we employed two A-terms with energy positions corresponding to the observed features in the absorption spectra. This indicates that the LUMO of these molecules generating the Q band is a formerly degenerated state undergoing Zeeman splitting in the magnetic field.

With respect to the spectroscopic ellipsometry and magneto-optical Kerr effect spectroscopy characterization of thin films of (metallo)porphyrin derivatives on opaque substrates, a few key features should be emphasized: The combination of this methods provides access to the intrinsic optical and magneto-optical constants of the (metallo)porphyrins and thereby to the nature of the optically induced electronic transitions. Furthermore, VASE can give an insight into structure and morphology of the films in terms of film thickness, surface roughness, as well as average orientation of the (metallo)porphyrin molecules in the films.

**Interplay of hydrogen bonding and molecule–substrate interaction in self-assembled adlayers of H_2_TPP(OH)_4_ on Au(111) and Ag(110)** [44]**:** To optimize the transport properties of potential electronic or spintronic devices it is necessary that the thin molecular films show a reproducible and stable arrangement with a high degree of long-range order. One way to improve the self-assembly is to use hydrogen bonding between, e.g., peripheral hydroxyl groups of the porphyrin molecules instead of the previously discussed molecules with OMe groups [30–32]. However for monolayer thin films, the substrate also plays a deciding role in the self-assembly, which will be reviewed in this section.

Thin films of the free-base porphyrin H_2_TPP(OH)_4_ were deposited by OMBD (pressure approximately 1 × 10^−8^ mbar, temperature around 350 °C) on Au(111) and Ag(110). The thin films were characterized by scanning tunneling microscopy (STM) experiments with a variable-temperature STM device. For further measurement details see [44].

**Figure 7:**
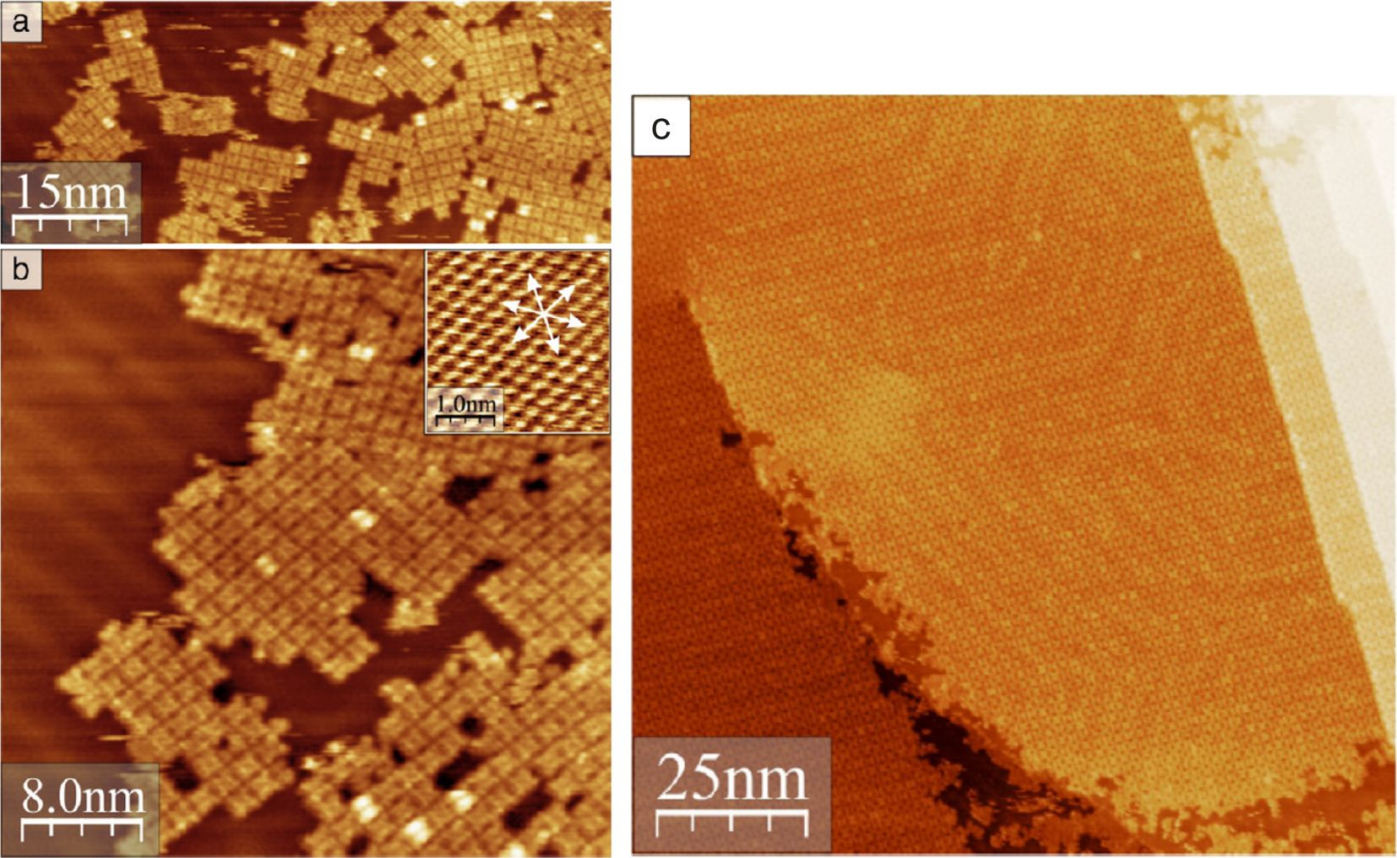
Results of STM measurements: (a,b) Formation of small islands at submonolayer coverage. Molecules on top of the first layer appear brighter (*U* = −1 V, *I* = 100 pA). Inset in (b): atomically resolved Au lattice (*U* = −0.5 V, *I* = 500 pA). Arrows are along the direction of the unit-cell vectors of the Au(111) substrate. (c) Large ordered domain of H_2_THPP (= H_2_TPP(OH)_4_) on Au(111) after annealing at ca. 150 °C (*U* = −1.5 V). Reproduced with permission from [44], copyright 2014 Elsevier.

On Au(111), and directly after initial adsorption, the formation of self-assembled small islands composed of several molecules of H_2_TPP(OH)_4_ became visible (Figure 7a,b). At a coverage of around 0.8 of a monolayer the individual islands appear rotated to each other by 120°, induced by the *C*_3_ surface symmetry of the Au(111) surface (Figure 7a,b). Upon annealing to around 150 °C highly ordered and large domains were observed, as shown in Figure 7c.

**Figure 8:**
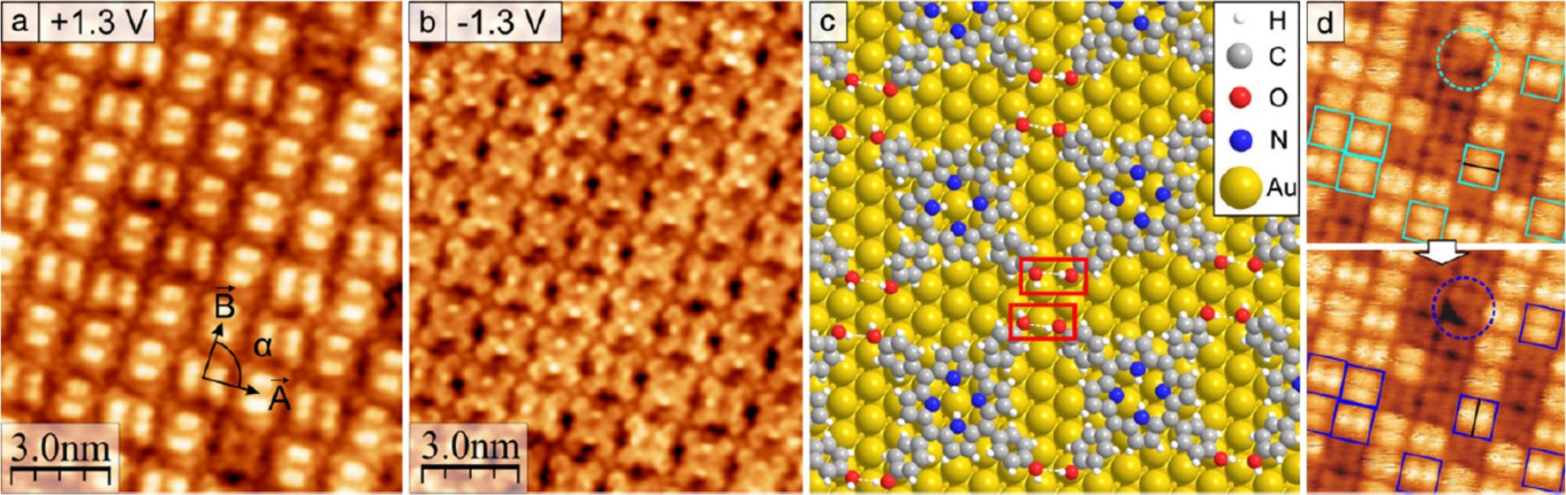
Highly resolved filled (a) and empty molecular states (b) STM image of the square structure of H_2_THPP (= H_2_TPP(OH)_4_) on Au(111). (c) Model for the arrangement of H_2_THPP (= H_2_TPP(OH)_4_) molecules in this structure. Pair-wise hydrogen bonding is marked with red rectangles. The reconstruction of Au(111) is not included in the model of the epitaxy. (d) Molecules which are rotated by 90° in the subsequently measured image (bottom) are marked by squares. A rotated molecule with one missing phenyl group is indicated by a dotted circle (11 nm × 10.5 nm, *U* = +1.2 V, sample annealed to ≈150 °C). Reproduced with permission from [44], copyright 2014 Elsevier.

O–H^…^O hydrogen bonds responsible for the formation of the well-ordered domains are shown exemplarily in Figure 8c. A tip-induced azimuthal 90° rotation of individual H_2_TPP(OH)_4_ molecules was observed, as revealed by two successive measurements and proven by the rotation of a defective molecule indicated with a circle in Figure 8d. A further remarkable feature of the adlayers on Au(111) was noticed: While measuring at lower absolute bias voltages (in-gap, see below) an identical appearance of the deposited molecules was found, whereas at higher absolute bias voltages two different appearances were noticed. The origin of this observation is discussed below, see [45].

**Figure 9:**
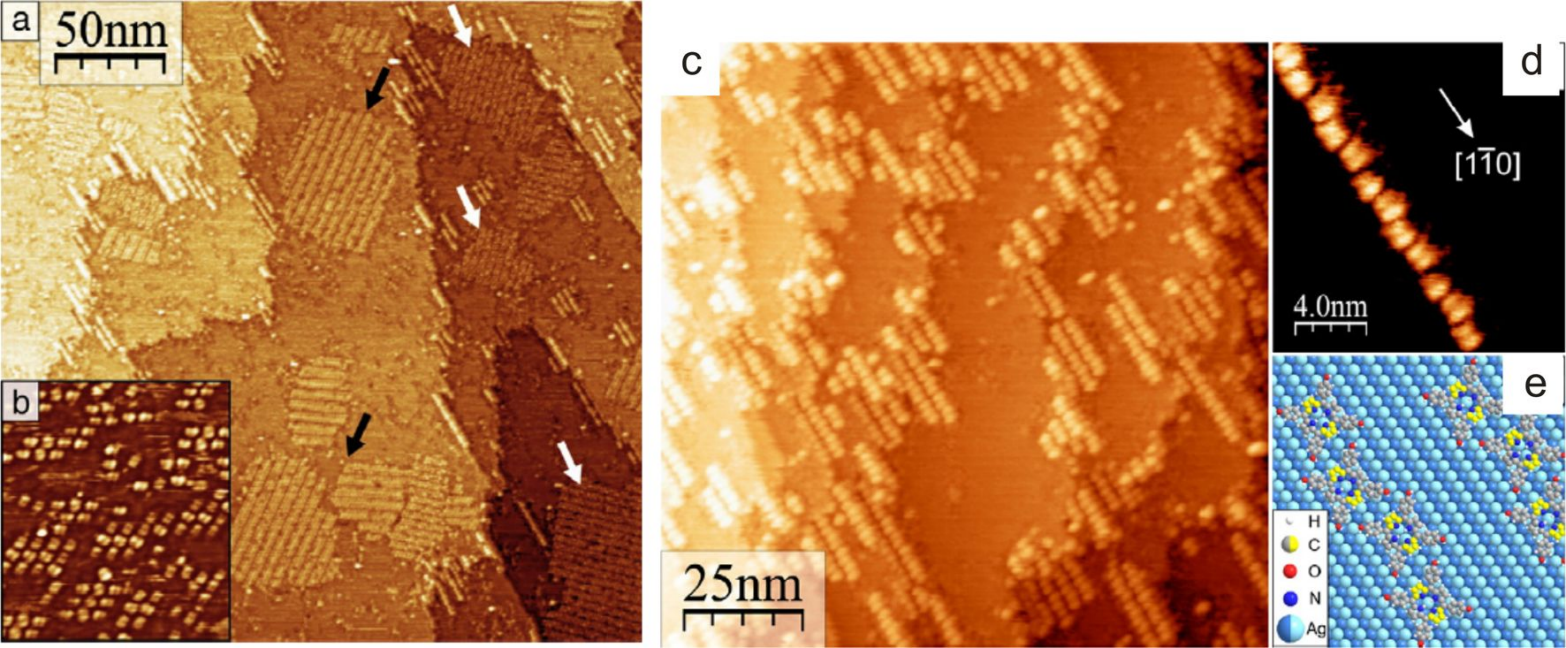
(a) Large scale STM image of submonolayer coverage showing molecular chains and ordered islands of H_2_THPP (= H_2_TPP(OH)_4_) on Ag(110) after annealing at about 200 °C (black/white arrows for the different 2D structures, *U* = +1.0 V). (b) Aggregation into small molecular islands after the first annealing at ca. 150 °C (23.1 nm × 26.2 nm, *U* = +1.4 V). (c) Molecular chains along 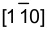 near step edges of Ag(110) (*U* = +0.7 V). (d) Magnification of a chain. H_2_THPP molecules are slightly displaced in a zigzag manner (*U* = +1.6V). (e) Model of epitaxy of the molecules in chains on Ag(110). Carbon atoms in the pyrrole rings bent away from the surface colored in yellow, Ag atom in first layer in bright blue, in second layer dark blue. Reproduced with permission from [44], copyright 2014 Elsevier.

In additional OMBD experiments, H_2_TPP(OH)_4_ was deposited on Ag(110). In contrast to observations made for the deposition on Au(111), see above, on Ag(110) and immediately after deposition of a submonolayer coverage only isolated molecules of H_2_TPP(OH)_4_ were observed [44]. This observation led to the conclusion that H_2_TPP(OH)_4_ has a lower diffusion activity on Ag(110) as compared to Au(111). Annealing at around 150 °C increased the mobility of the adsorbed molecules and resulted in the formation of small islands composed of nine or twelve molecules of H_2_TPP(OH)_4_ (Figure 9a). Subsequent heating to around 200 °C had the molecules rearrange into molecular chains along 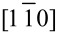 near step edges of Ag(110) (Figure 9c), striped islands and differently ordered areas as shown in Figure 7a,b. For further details about the self-assembled ensembles of H_2_TPP(OH)_4_ on Ag(110), including illustrations of the hydrogen bonds that are responsible for interaction of the molecules with each other and accompanying density functional theory calculations, we refer to [44].

This work led to the conclusion that H_2_TPP(OH)_4_) forms a nearly complete monolayer on Au(111) and that this single-crystalline metallic surface does not have major influence on the self-assembly. In contrast, on the highly anisotropic Ag(110) surface the observed self-assembled structures of H_2_TPP(OH)_4_) can be related to the number of hydrogen bonds and the occupation of favorable adsorption positions. Moreover, molecule–substrate interactions are more pronounced on Ag(110) compared to Au(111).

**Manipulation of the electronic structure of H_2_TPP(OH)_4_ on Au(111)** [45]**:** Molecules with two possible states, e.g., of conductivity, can be used as single-molecule switches. This functionality could be applied in nano-scaled molecular-based memory devices or logic gates [64], However, one must be able to switch reversibly and controllably between the two stable states. An interconversion between different electronic or magnetic properties can be obtained, e.g., by inducing conformational or configurational changes in a molecular system [65], or by binding or releasing small molecules or atoms [66–67].

**Figure 10:**
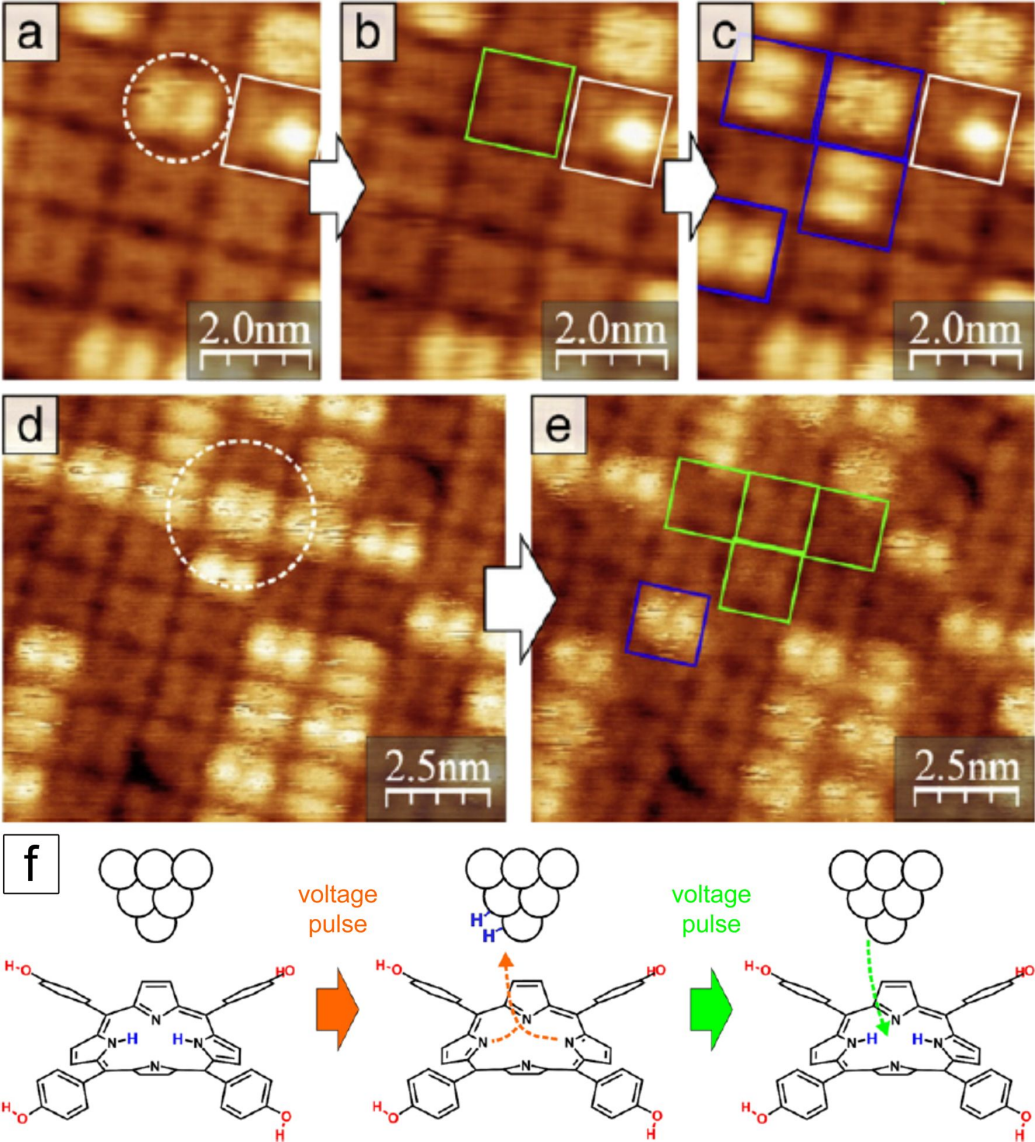
(a–e) Manipulation of the electronic structure by applying a voltage pulse with the STM tip at the position marked with a white circle. Converted molecules are marked with green (1→2) or blue (2→1) rectangles. (a–c) 1→2→1 conversion with 2 V pulses for 3 s (feedback switched on) at the same position before each frame. A molecule with only one protrusion (white rectangle) is used to mark the position in the scanned area. (d,e): Changing of neighboring molecules in both directions during the same pulse of 2.5 V for 3 s imaging parameter: +1.2 V, 100 pA. (f) Scheme of the proposed transfer of 2H from H_2_TPP (= H_2_TPP(OH)_4_) to the tip apex and from the tip back to the dehydrogenated molecule by voltage pulses (1→2→1). Reproduced with permission from [45], copyright 2014 Elsevier.

As described above and in [44], an identical in-gap appearance but two different appearances of the deposited H_2_TPP(OH)_4_ molecules at a higher bias voltage was observed. In order to further investigate this phenomenon, H_2_TPP(OH)_4_ was deposited again by OMBD on Au(111) and subsequently annealed at 150 °C for one hour to achieve uniform coverage [44–45]. The voltage dependence of the appearance of the molecules in STM was investigated and it was realized that bias voltages of larger than 0.7 V were required in order to clearly observe H_2_TPP(OH)_4_ in two different states, denoted as 1 and 2. Scanning tunneling spectroscopy measurements enabled us to determine the HOMO–LUMO gap of 1 to be 2.0 ± 0.1 and of 2 to be 2.5 ± 0.1 eV. If the STM is measured inside the HOMO–LUMO gaps, geometry effects dominate and all molecules in the ordered layer look quite identical. This changed when bias voltages higher than 1.5 V were applied and also a sudden conversion between 1 and 2 was observed. It is possible to control the interconversion between 1 and 2 by the STM tip. A typical case is shown in Figure 10a–e, where the tip is positioned over a molecule and the only the bias voltage is modified. The interconversion events are reversible and are attributed to hydrogen transfer from H_2_TPP(OH)_4_ to the tip and back (Figure 10f). We demonstrated that it is possible to induce the formation of state 2 spatially (Figure 10a–e) and electronically (above 2 V) well resolved for individual molecules by using voltages between 1.5 and 2 V while higher voltages also switch neighboring molecules. In addition, states 1 and 2 are part of a large and highly ordered self-assembled array and it is possible to read-out the conductance at a specified position, and thus state 1 or 2, in a non-manipulative manner. Furthermore, it is possible to reverse positions of 1 and 2. Due to these unique features it seems possible to apply them for the construction of a nano-scale memory device.

**Figure 11:**
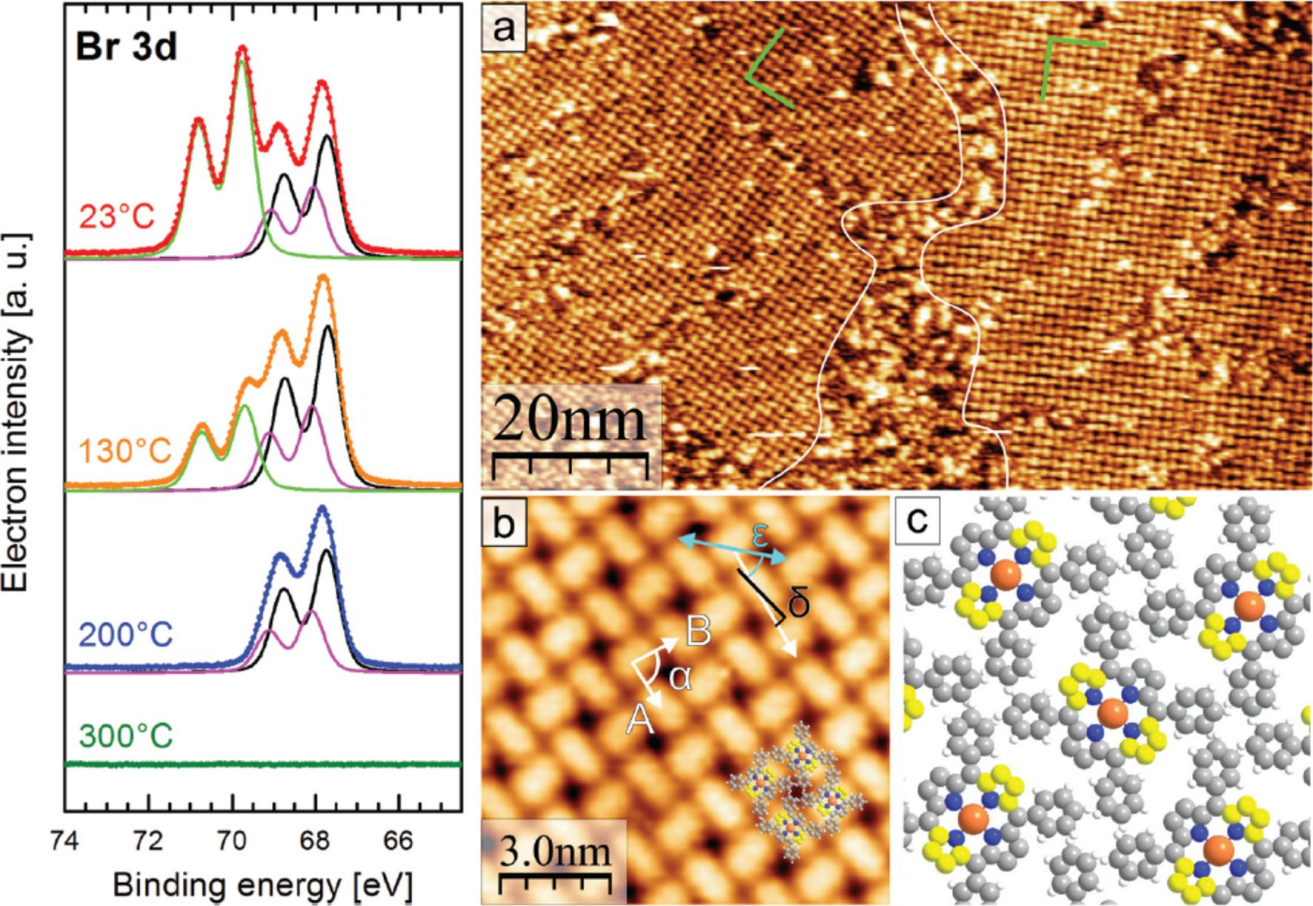
Left: Evolution of the XPS spectra of Br 3d for a molecular monolayer of CuTPP(Br)_8_ on Au(111) as a function of the indicated annealing temperature. In the Br 3d spectra, the background has been subtracted. Right: (a) STM image after deposition of ca. 1 ML of CuTPP(Br)_8_ and annealing at max. 200 °C. Two periodically ordered rotational domains and disordered areas in between are shown. The herring-bone reconstruction of the underlying Au(111) is visible through the large ordered areas (*U* = −0.7 V). (b) Magnification of the adlayer structure of CuTPP(Br)_8_ on Au(111) with elongated appearance of molecules at *U* = −1 V. The cyan arrow indicates the direction of one unit cell vector of the Au(111) surface lattice. (c) Model of the molecular arrangement; protruding C atoms of the saddle deformation are colored yellow. Adapted from [47] with permission from The Royal Society of Chemistry.

**Surface-confined 2D polymerization of CuTPP(Br)_8_ on Au(111)** [47]**:** Covalent linking of organic molecules directly on the surface would allow for the engineering of manifold extended 2D materials with novel transport properties. One major approach for surface-confined polymerization is the halogen-based Ullmann coupling reaction [42]. Thereby, the topography and also electronic properties of the covalent organic framework are determined by especially the halogen substitution pattern of the monomer. Notably, probably the first example of halogen-based, two-dimensionally covalent self-assembly on a surface was demonstrated by Grill and co-workers [40], where they showed that dimers, oligomer chains and small 2D covalent networks could be formed from porphyrin molecules with different numbers of bromine substitutions. The properties of the substrate surface have a strong influence in this kind of on-surface polymerization reaction. A high reactivity and also high energy barriers for diffusion can result in immediate C,C coupling after dehalogenation of the monomers. So far most 2D polymers that were reported are diffusion-limited and therefore have a small domain sizes, a high density of defects, or the networks are even undesiredly crumpled and without long-range order [68–70]. To solve this problem a coupling-limited polymerization [39,41], in which the radical molecules can diffuse and arrange before they couple irreversibly with each other, is needed. One example of this is summarized in the following. OMBD at around 350 °C was used to deposit approximately one monolayer of CuTPP(Br)_8_ on a Au(111) wafer kept at room temperature. X-ray photoelectron spectroscopy (XPS) measurements indicated that after initial deposition not all CuTPP(Br)_8_ molecules remained intact (Figure 11, left column). Likely, some molecules partially debrominate during OMBD in the crucible or when arriving with high thermal energy on the Au(111) surface. Sample annealing at only 130 °C reduced the number of intact CuTPP(Br)_8_ molecules on a Au(111) further, at 200 °C only surface-adsorbed Br atoms or Br_2_ molecules were observed and at 300 °C all bromine species desorbed (Figure 11, left column).

**Figure 12:**
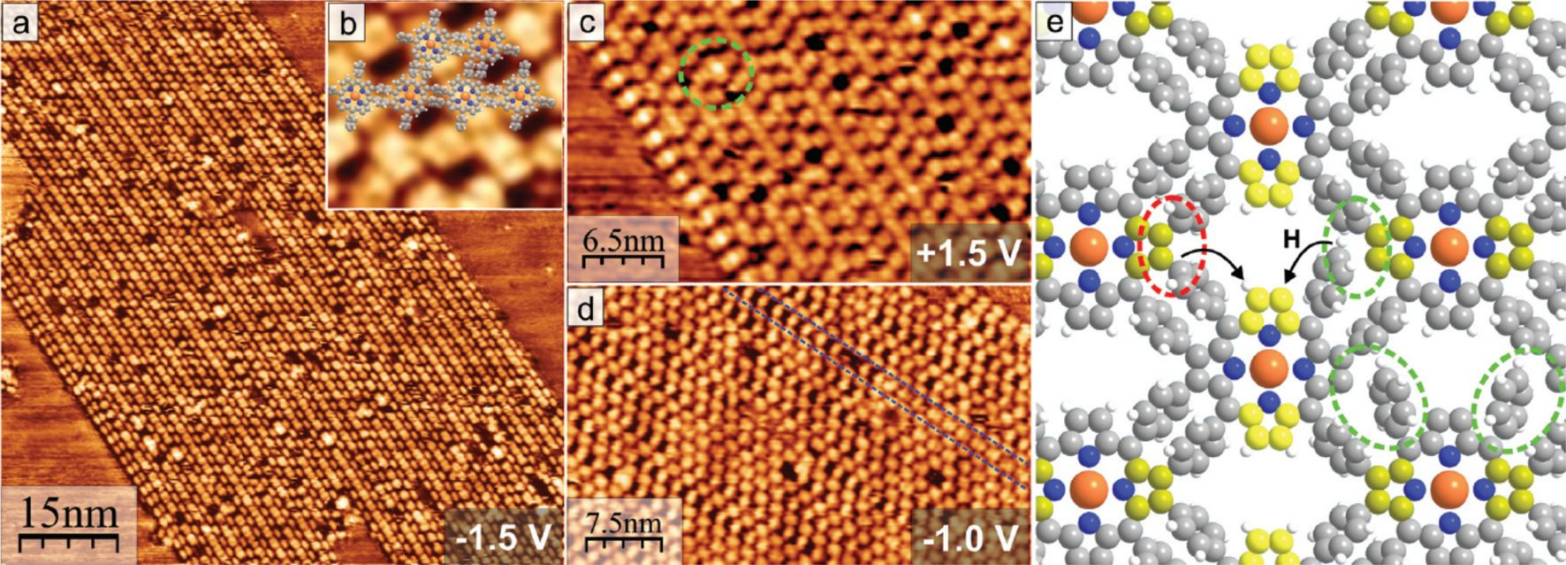
Results of STM measurements: (a) Novel structure of the alternating molecular rows formed after annealing at 350 °C (*U* = −1.5 V). (b) Overlay of molecular model on the structure (*U* = +1.5 V, 6.5 nm × 6.5 nm). (c) Image of the occupied molecular orbitals at the left side of the ordered structure shown in (a). The green circle marks a lattice defect. (d) Defect along molecular rows (marked blue) from the incomplete C,C-coupling between neighboring molecules. (e) Model of the arrangement with covalent bonds between the debrominated molecules (red circle). Incomplete polymerization is marked by the green circle. Dissociated hydrogen atoms from the phenyl groups could diffuse and bond to the still unsaturated carbon atoms (arrows). Upwards directed C atoms in the pyrrole units of the porphyrin saddle shape are colored in bright and dark yellow to indicate slightly different angles as explained in the text. Adapted from [47] with permission from The Royal Society of Chemistry.

STM measurements were carried out in order to determine surface structures of deposited CuTPP(Br)_8_ on a Au(111) surface. After deposition of about one monolayer only small periodically structured arrangements were observed. Annealing significantly enlarged the size of ordered areas, which is attributed to a remarkable surface mobility [71] of the adsorbed molecules at increased temperature. At a temperature of 200 °C most of the surface was covered with ordered areas, although some disordered regions were noticed (Figure 11a–c). As depicted in Figure 11c the imaged molecules correspond to radical CuTPP, as XPS measurements demonstrated for that temperature the presence of surface-bound Br/Br_2_ only. Interestingly, these surface-stabilized radicals of CuTPP are seemingly long-term stable. The result obtained after heating this sample to 350 °C for one hour is displayed in Figure 11. It resulted in the formation of nano-ribbons (Figure 12) and is accompanied by a coverage reduction of about 25%. Likely, the coverage reduction is due to partial desorption and reassembling, although it remains speculative at the present time to what extent. The STM picture of one nano-ribbon is shown exemplarily in Figure 12. Obviously, several hundred CuTPP species are self-assembled into an at least 75 × 45 nm large ribbon, representing a covalently bonded network of CuTPP. These astonishingly large dimensions of the nano-ribbons might be attributed to several reasons. For example, the surface polymerization could be shown to proceed stepwise, that is, all bromine species were fully cleaved-off before the Ullmann-type C,C-coupling reactions between neighbored porphyrin stages were initiated [42]. Furthermore, the surface-mobility of adsorbed CuTPP(Br)_8_ and CuTPP species is obviously sufficiently high to allow for reassembling. To the best of our knowledge such a relatively large-scale 2D on-surface polymerization of porphyrin molecules has been observed here for the first time. As illustrated in Figure 12, the nano-ribbon comprises several coupling defects as typically observed for covalent networks. Four different types of such defects have been thoroughly discussed by us together with possibilities to avoid or to cure them.

## Conclusion

We have described our investigation of thin films of (metallo)porphyrins deposited by OMBD with particular emphasis on their structural, morphological, (magneto-)optical and electronic properties. We showed a few recipes for functionalization of (metallo)porphyrins on how to assemble individual molecules further into large non-covalently and covalently bonded ensembles. The potential for applications of the results reviewed here ranges from dendrites as nanowires for electronic/spintronic device integration via self-assembled non-covalently locked 2D layers for nano-scaled memory devices to spintronic devices with laterally conductive and magnetic 2D nano-ribbons.

Of course, this is a typical status report that reflects the present state of knowledge acquired. However, the local transport techniques implemented here for the (metallo)porphyrin thin films represent a formidable approach in order to downscale and unveil electrical characteristics, which would certainly drive device performance. For the future the synthesized (metallo)porphyrins and methods used and applied have to be combined even further, whereby especially the combination of structural and (local) spectroscopic investigations is highly promising. For example, we regard the 2D nano-ribbons made of CuTPP(Br)_8_ molecules as a good starting point to investigate how to functionalize the porphyrins further with different (transition) metal atoms or functional groups to tailor structures and physical properties. The electronic, electrical and local magnetic properties of new 2D nano-ribbons will be investigated by, for example, cs-AFM or in situ four-probe STM. It is both challenging and motivating to investigate how to separate the 2D nano-ribbons from surfaces and to build multilayer devices from them. Thus, the results presented here gave plenty of stimuli to continue our joint efforts [60,72].

Finally, we would like to express our hope that the multifaceted fabrication and investigation methods of ensembles of (metallo)porphyrins, as different as they might appear, may stimulate joint approaches of material scientists to further explore their application potential.
